# Supplementary material for: Hybrid Models and Biological Model Reduction with PyDSTool
Source: PLoS Comput Biol. 2012 Aug 9;8(8):e1002628. doi: 10.1371/journal.pcbi.1002628 (PMC3415397; doi:10.1371/journal.pcbi.1002628)
Supplement: Text S4 — Complete source code for the PyDSTool package (version 0.88.120504). Includes API documentation and help files linking to web pages. This file is identical to the current public release on Sourceforge.net. (ZIP) [file pcbi.1002628.s004.zip › PyDSTool/html/PyDSTool.Toolbox.phaseplane.zone_node-class.html]

xml version="1.0" encoding="ascii"?


PyDSTool.Toolbox.phaseplane.zone\_node


| Home | Trees | Indices | Help | PyDSTool |
| --- | --- | --- | --- | --- |

Package PyDSTool ::
Package Toolbox ::
Module phaseplane ::
Class zone\_node

[hide private]

[frames] | no frames]

# Class zone\_node

source code

```
       object --+            
                |            
 MProject.feature --+        
                    |        
MProject.feature_node --+    
                        |    
 MProject.ql_feature_node --+
                            |
                           zone_node
```

Known Subclasses:
:   - nullcline\_zone\_node

---

Phase plane 'zone' node of hierarchical qualitative feature abstract
class. A zone is a bounding box in the phase plane containing a feature,
grown to include a region that satisfies some local condition.

Must define \_leaf\_class for each implementation of a zone\_node
class


|  |  |
| --- | --- |
| Instance Methods | [hide private] |

|  |  |
| --- | --- |
| finish(self, target)  Operations to complete only if evaluate was True (optional). | source code |

|  |  |
| --- | --- |
| \_local\_init(self) | source code |

**Inherited from `MProject.feature_node`**:
`__getitem__`,
`__init__`,
`__repr__`,
`__str__`,
`evaluate`,
`propagate_verbosity`,
`set_ref_traj`

**Inherited from `MProject.feature_node`** (private):
`_residual_info`

**Inherited from `MProject.feature`**:
`__call__`,
`__eq__`,
`__hash__`,
`__ne__`,
`info`,
`postprocess_ref_traj`,
`prepare`,
`reset_metric`,
`validate`

**Inherited from `MProject.feature`** (private):
`_find_idx`

**Inherited from `object`**:
`__delattr__`,
`__getattribute__`,
`__new__`,
`__reduce__`,
`__reduce_ex__`,
`__setattr__`


|  |  |
| --- | --- |
| Properties | [hide private] |

**Inherited from `object`**:
`__class__`


|  |  |
| --- | --- |
| Method Details | [hide private] |

|  |  |
| --- | --- |
| finish(self, target) | source code |

Operations to complete only if evaluate was True (optional). Override
in concrete sub-class if desired

Overrides: MProject.feature.finish: *(inherited documentation)*

  


| Home | Trees | Indices | Help | PyDSTool |
| --- | --- | --- | --- | --- |

|  |  |
| --- | --- |
| Generated by Epydoc 3.0.1 on Fri May 4 15:24:10 2012 | http://epydoc.sourceforge.net |
